# Supplementary material for: Friction-driven membrane scission by the human ESCRT-III proteins CHMP1B and IST1
Source: Proc Natl Acad Sci U S A. 2022 Jul 11;119(29):e2204536119. doi: 10.1073/pnas.2204536119 (PMC9303997; doi:10.1073/pnas.2204536119)
Supplement: Supplementary File [file pnas.2204536119.sapp.pdf]

PNAS

Supplementary Information for

Friction-driven membrane scission by the human ESCRT-III proteins CHMP1B and IST1

A. King Cada[1,7], Mark R. Pavlin[2,7], Juan P. Castillo[3,7,11], Alexander B. Tong[3,7], Kevin P. Larsen[1,7], Xuefeng Ren[1,7], Adam L. Yokom[1,7], Feng-Ching Tsai[8], Jamie V. Shiah[1,7], Patricia M. Bassereau[8], Carlos J. Bustamante[1-7,10], James H. Hurley[1,2,7,9,10]

Paste corresponding author name here
Email: carlosb@berkeley.edu or jimhurley@berkeley.edu

This PDF file includes:

Figures S1 to S4
Legends for Movies S1 to S7

Other supplementary materials for this manuscript include the following:

Movies S1 to S7

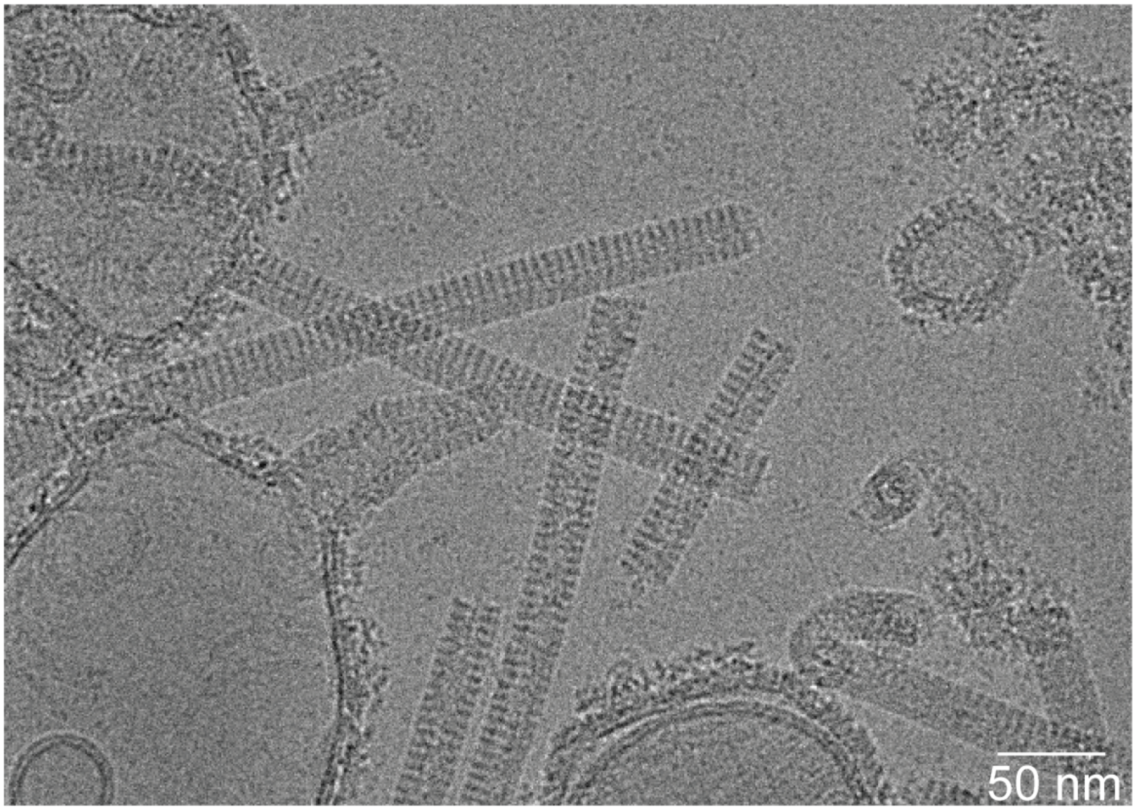

Fig. S1. Representative cryo-electron micrograph of CHMP1B and IST1^{NTD} tubulating and constricting the membrane.

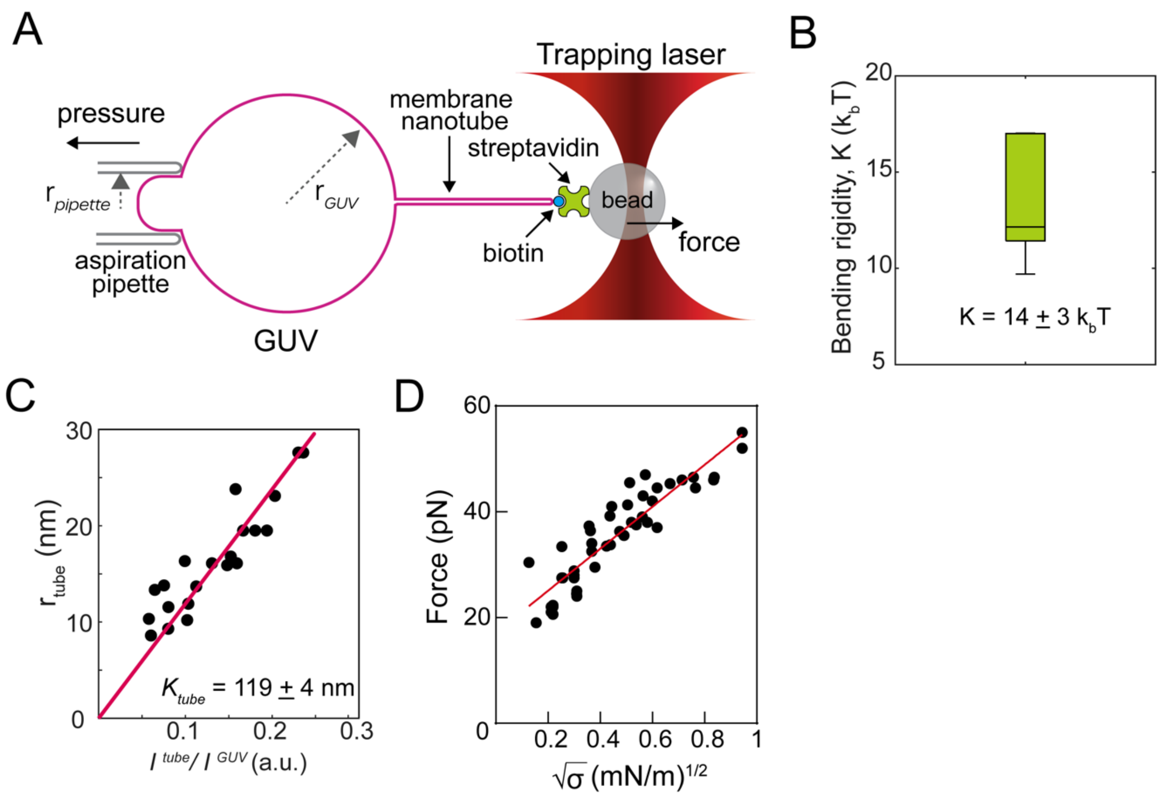

Fig. S2. Tube radius measurement. (*A*)**,** Schematic of tube radius, in the absence of proteins, using an optical trap to extract force and membrane tension, σ, from the relationship $\sigma = \frac{\Delta P \times r_{pipette}}{2(1 - r_{pipette}/r_{GUV})}$, where $r_{pipette}$ is the pipette radius, r_{GUV} is the GUV radius, and ΔP is the difference in aspiration pressure. (*B*)**,** Bending rigidity, K, is determined to be 14 ± 3 k_bT by plotting the linear relationship between force and $\sqrt{\sigma}$. (*C*)**,** We determined tube radii measurements using the ratio between the fluorescence intensity of the GUV, I^{tube} and the fluorescence intensity of the membrane nanotube, I^{GUV} multiplied by a constant K_{tube} as described in (47). K_{tube} is experimentally determined by plotting the tube radius of a bare GUV (*N*=9) calculated from the expression r_{tube} = F/4πσ, where σ is the membrane tension and F is the force measured using the optical trap and plotting against I^{tube}/I^{GUV}. We find the calibration constant K_{tube} = 119 ± 4 nm. (*D*) Tube retraction force as a function of membrane tension $\sqrt{\sigma}$.

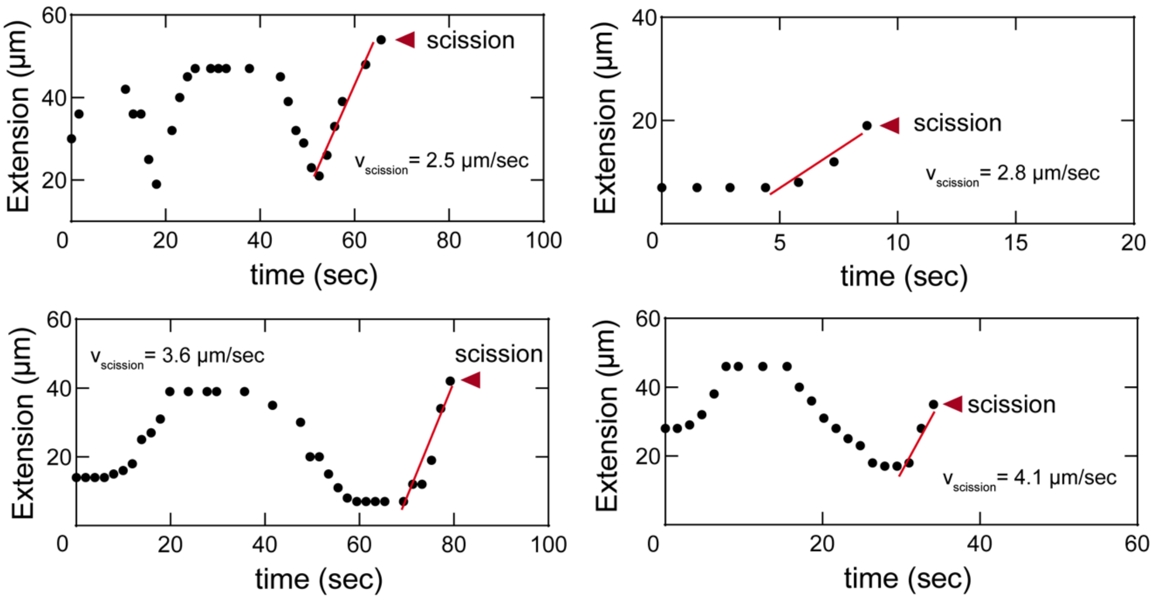

Fig. S3. Additional extension plots of all tube pulling trials.

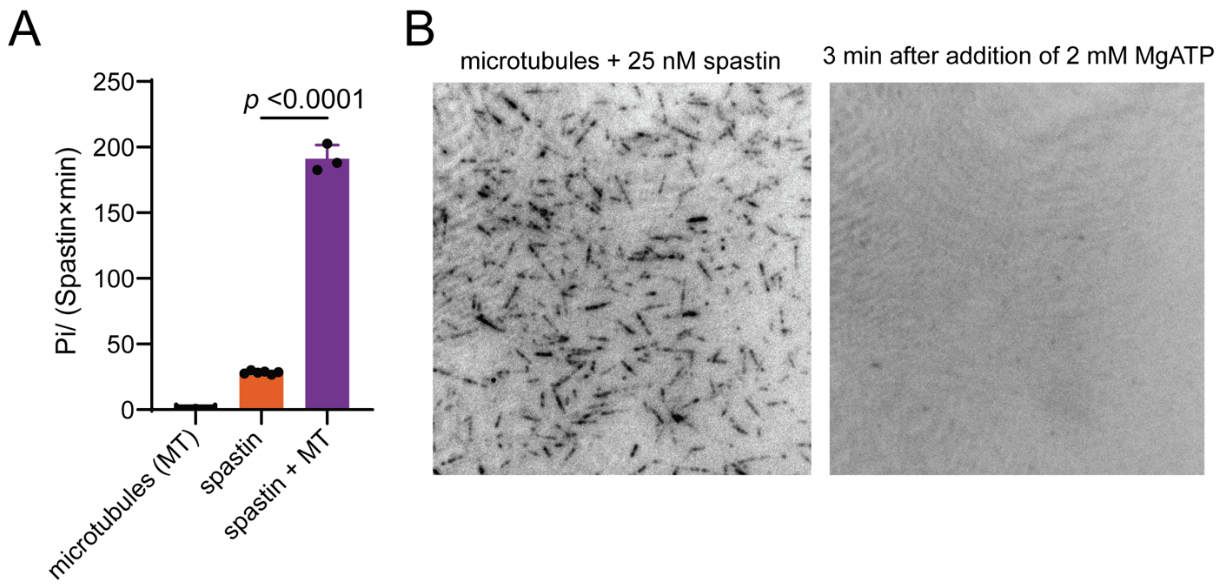

Fig. S4. Spastin disassembles microtubules in an ATP-dependent manner. (*A*) Spastin activity is stimulated in the presence of microtubules (191 ± 10 ATP/spastin·min). (*B*) Fluorescently-labeled microtubules are immobilized on a coverglass together with spastin. Addition of ATP disassembles microtubules.

Movie S1 (separate file): Tube elongation in the absence of protein scaffold. Bare membranes pulled at > 25 $\mu m.s^{-1}$ at 0.2 $pN.nm^{-1}$ do not break even after repeatedly being brought back-and-forth. Movie plays in real time.

Movie S2 (separate file): External pulling on protein-scaffolded tubes promotes scission. Membrane tube pulled at 3 $\mu m.s^{-1}$ on membrane bound LD555-CHMP1B-$IST1^{NTD}$ (green) protein scaffolds induces scission. Black arrow highlights the point of scission. Movie plays at 15x real time.

Movie S3 (separate file): Spastin colocalizes with ESCRT-III without uncoating or severing the tube. 5 μM LD555-CHMP1B and 5 μM $IST1^{NTD}$ were dispensed using a micropipette in proximity to the region of interest. 5 μM Spastin with 1 mM ATP was added after LD555-CHMP1B fluorescence equilibrated. LD655- spastin (cyan) colocalizing on LD555-CHMP1B (green) and $IST1^{NTD}$ enriched sites on the membrane (magenta). Movie plays at 25x real time.

Movies S4-S7 (separate files): Additional replicates of the scission experiment shown in Movie S2. Movies play at 65x real time and terminate after the scission event.
